# Adaptive inter-tissue proteostasis networks in aging and neurodegeneration

**DOI:** 10.1042/BSR20254097

**Published:** 2026-01-09

**Authors:** Carlos A. Vergani-Junior, Matheus Antonio V. de C. Ventura, Evandro A. De-Souza

**Affiliations:** 1 Departamento de Bioquímica, Instituto de Química, Universidade de São Paulo, São Paulo, Brazil

**Keywords:** proteostasis network, unfolded protein response (UPR), heat shock response (HSR), cell-non-autonomous signaling, aging

## Abstract

The maintenance of proteostasis is essential for cellular function and organismal health. Its decline with age is a key contributor to neurodegenerative diseases, metabolic disorders, and other chronic conditions. Eukaryotic cells respond to proteotoxic stress through compartment-specific pathways, including the heat shock response (HSR), the unfolded protein response of the endoplasmic reticulum (UPR^ER^), and the mitochondrial UPR (UPR^mt^). While these pathways have been extensively studied in cell-autonomous contexts, recent evidence reveals that neurons and glial cells can co-ordinate these responses across tissues through cell-non-autonomous mechanisms. Neuronal signals, including neuropeptides, biogenic amines, and possibly extracellular vesicles, can activate stress responses in distal cells, modulating lipid metabolism and impacting longevity. Emerging data also suggest a role for glial cells in systemic proteostasis regulation, though their mechanisms remain relatively uncharacterized. This review discusses both classical and emerging concepts of proteostasis stress-response pathways, their integration with neural signaling, and how their modulation influences aging and disease. Understanding how intercellular communication governs proteostasis could open new avenues for therapeutic interventions in age-related and neurodegenerative disorders.

## Introduction

Proteostasis, or protein homeostasis, refers to the finely tuned co-ordination of a protein’s life cycle, including its protein synthesis, folding, trafficking, and degradation [1]. This complex regulation is essential for maintaining cellular integrity and ensuring resilience to diverse physiological stresses such as thermal, oxidative, and immune challenges [1]. Comparative studies highlight that while prokaryotes possess basic proteostasis components such as chaperones and proteases, eukaryotes present specialized stress-response pathways that manage the greater folding burden of a larger, more diversified proteome [2]. This expansion is particularly evident in multicellular organisms, where inter-tissue co-ordination of proteostasis becomes critical. Yet, although multicellular organisms rely on stress-response pathways to preserve proteostasis across tissues, these protective mechanisms become less efficient with age. Indeed, the loss of proteostasis is considered one of the 12 hallmarks of aging [3]. As organisms age, they lose the capacity to maintain proper function of mechanisms involved in protein synthesis, folding, quality control, and degradation [3]. This decline in proteostasis often results in the accumulation of misfolded and aggregated proteins, a characteristic of many neurodegenerative diseases such as Alzheimer’s disease (AD), Parkinson’s disease (PD), and Huntington’s disease (HD) [4], but also in other chronic conditions, including cancer, obesity, and metabolic syndromes [5].

To maintain the proteome integrity, eukaryotes have evolved highly compartmentalized, adaptive, and specialized stress-response mechanisms. The cytosol engages the heat shock response (HSR), while the mitochondria and the endoplasmic reticulum (ER) activate specific branches of the unfolded protein response (UPR), namely the mitochondrial UPR (UPR^mt^) and the UPR of the ER (UPR^ER^), respectively [6]. The activation of these pathways usually relies on chaperones sensing misfolded proteins in an autonomous way, triggering signaling cascades that activate transcription factors. Although cell-intrinsic activation mechanisms are well characterized, cross-organelle signaling, cell-non-autonomous communication, and metabolic integration are increasingly recognized as key regulators of the proteostasis network. In the sections below, we will highlight key branches of the proteostasis network that are most directly implicated in aging and age-related diseases. We will then explore how modulation of these proteostasis pathways influences aging within the nervous system through cell-autonomous mechanisms, as well as in peripheral tissues via cell-non-autonomous signaling.

### The HSR

The HSR is a highly conserved cellular defense mechanism that safeguards proteome integrity in the face of cytosolic stress. First discovered in flies [7,8], the HSR was initially identified as a transcriptional response to elevated temperatures, leading to the synthesis of a specific set of proteins, now known as heat shock proteins (HSPs). The accumulation of misfolded or aggregated proteins, particularly HSP70, which normally sequesters the transcription factor heat shock factor 1 (HSF1) in an inactive monomeric form [9]. During proteotoxic stress, HSP70 is titrated away, allowing HSF1 to trimerize, translocate to the nucleus, and bind heat shock elements to drive the expression of chaperones (e.g. HSP70, HSP90). In mammals, HSF1 activity is regulated by posttranslational modifications, including phosphorylation and acetylation, which modulate its transcriptional activity [9]. For a comprehensive overview of HSR molecular kinetics and regulation, we refer readers to comprehensive reviews [9,10].

Besides thermal stimuli, the HSR can be activated by mitochondrial signals. It was demonstrated that mitochondrial electron transport chain (ETC) dysfunction during development in *Caenorhabditis elegans* activates cytosolic HSF-1 via PP2A-mediated dephosphorylation and promotes the expression of HSPs in adulthood, protecting against protein aggregation [11,12]. Beyond its role in stress adaptation, the HSR pathway has been implicated in longevity and age-related diseases. In model organisms, enhanced HSR activity correlates with extended lifespan, while age-associated decline in HSF1 function was described, and it has been linked to organismal physiological decline [13–15]. Furthermore, dysregulation of the HSR is observed in some neurodegenerative disorders characterized by protein misfolding, such as PD and HD [16,17].

### UPR of the endoplasmic reticulum

The UPR^ER^ is a conserved adaptive mechanism activated in response to ER stress, which can result from the accumulation of misfolded proteins or perturbations in membrane homeostasis [18]. The response is mediated by three main transmembrane sensors: IRE1, PERK, and ATF6. The detailed signaling cascades of these branches are extensively reviewed elsewhere [18–20] and are briefly summarized here.

The most conserved branch involves IRE1, a kinase/endoribonuclease that senses stress through its luminal domain or membrane properties [21]. Under homeostatic conditions, IRE1 is kept inactive via association with the ER chaperone BiP (GRP78). Upon detecting luminal stress (via BiP dissociation) or lipid bilayer stress, IRE1 catalyzes the non-canonical splicing of XBP1 mRNA. XBP1s translocates to the nucleus and drives the expression of genes involved in ER protein folding (e.g. chaperones), ER-associated degradation, and lipid biosynthesis, collectively restoring ER function [18]. Distinct from this splicing event, IRE1 can also degrade ER-localized mRNAs through regulated IRE1-dependent decay (RIDD) to reduce the protein influx [22,23]. Substrate selection by RIDD appears to rely on both sequence motifs and subcellular localization, often involving consensus stem-loop structures resembling those in XBP1 mRNA [24]. Physiologically, RIDD exerts a cytoprotective effect under mild stress by reducing ER load; however, under chronic or excessive stress, its activity may trigger apoptosis [23,25,26].

The second arm of the UPR^ER^ is mediated by the ER transmembrane kinase PERK (PKR-like ER kinase). Upon ER stress, PERK phosphorylates the translation initiation factor eIF2α [19]. This phosphorylation event reduces general cap-dependent translation but enhances the translation of specific transcripts with regulatory upstream open reading frames [18], such as ATF4, which regulates genes involved in redox control, amino acid metabolism, autophagy, and apoptosis, including CHOP, a key pro-apoptotic factor under continuous stress [19]. Importantly, phosphorylation of eIF2α is not unique to PERK activation but constitutes the core of the integrated stress response (ISR), a convergent pathway that modulates translation in response to diverse intracellular stressors [19]. In addition to ER stress-induced PERK, three other kinases, namely GCN2 (activated by amino acid deprivation), PKR (activated by viral double-stranded RNA), and HRI (activated by heme deficiency or oxidative stress) can phosphorylate eIF2α.

Finally, the ATF6 branch involves the translocation of the sensor to the Golgi apparatus upon stress through a proteolytic cleavage, releasing a cytosolic fragment of ATF6 [27,28]. The liberated cytosolic fragment enters into the nucleus and activates transcription of ER quality control genes and chaperones. Notably, ATF6 also induces XBP1 expression, thereby amplifying the IRE1 pathway [29].

### Mitochondrial UPR

The mitochondria also possess a proteostasis surveillance system. In animals, this compartment-specific response is known as the mitochondrial UPR. It is a conserved cellular pathway that safeguards mitochondrial proteostasis and function when challenged by protein misfolding, oxidative damage, or impaired import of nuclear-encoded proteins. Although early evidence for a mitochondrial stress response was observed in mammalian cells [30], the pathway was mechanistically elucidated in the nematode *C. elegans* through genetic studies [31,32]. These studies revealed that mitochondrial dysfunction elicits a distinct nuclear transcriptional program independent of the UPR^ER^, yet mechanistically reminiscent in its use of retrograde signaling. For an in-depth analysis of UPR^mt^ mechanisms and their evolutionary conservation, we direct readers to the UPR^mt^-focused reviews [33,34].

Mechanistically, the UPR^mt^ is activated by mitochondrial stressors such as protein misfolding, loss of membrane potential, or excessive reactive oxygen species (ROS). The pathway is regulated by the transcription factor ATFS-1 (in *C. elegans*) or ATF5 (in mammals), which contains both a mitochondrial targeting sequence and a nuclear localization sequence. Under homeostatic conditions, ATFS-1 is efficiently imported into the mitochondrial matrix and degraded by the protease LONP-1. However, during stress, reduced membrane potential or chaperone saturation compromises this import. Consequently, ATFS-1 escapes degradation and accumulates in the nucleus. Concurrently, the mitochondrial protease ClpP degrades misfolded proteins into peptides that are exported to the cytosol via the transporter HAF-1, acting as a complementary retrograde signal [35–37]. Once in the nucleus, ATFS-1 co-operates with the chromatin remodeler DVE-1 and the ubiquitin-like protein UBL-5 to drive the expression of mitochondrial chaperones (e.g. HSP-60), proteases, and ROS detoxification enzymes [35].

In addition to restoring mitochondrial proteostasis, the UPR^mt^ promotes mitochondrial biogenesis and activates innate immunity genes via cross-talk with the p38 MAPK and innate surveillance pathways [35,38]. Posttranslational regulation fine-tunes this response. SUMOylation was shown to stabilize ATFS-1, while the SUMO-specific protease ULP-4 modulates both ATFS-1 activity and DVE-1 nuclear localization, adding another regulatory layer to the UPR^mt^ [39]. The UPR^mt^ is implicated in aging and neurodegeneration, where persistent mitochondrial stress may chronically activate or dysregulate the UPR^mt^, contributing to pathology [40,41], although the activation of UPR^mt^ does not seem to be sufficient to drive lifespan extension alone [42].

### Neural cell-non-autonomous regulation of proteostasis pathways in physiology

While the pathways described above were historically characterized as cell-intrinsic mechanisms – where a cell detects and resolves its own stress – seminal discoveries in *C. elegans* have fundamentally shifted this view. It is now established that tissues, especially the nervous system, can co-ordinate these responses across distal tissues, allowing the organism to anticipate challenges and synchronize metabolic states.

This paradigm was first established for the HSR by Prahlad and colleagues, who demonstrated that thermosensory neurons (AFD) are required to activate the HSR in somatic tissues, even when those tissues are not directly exposed to heat [43]. Subsequently, a similar neuroendocrine axis was identified for the mitochondria, where neuronal stress triggered the UPR^mt^ in the intestine, a response sufficient to extend lifespan [44]. Crucially, this systemic control extends to the ER with distinct mechanistic requirements. In a seminal study, Taylor and Dillin demonstrated that the age-related decline of the UPR^ER^ is reversible: specific expression of spliced *xbp-1s* in neurons constitutively activates the UPR^ER^ in the intestine and extends lifespan. This inter-tissue co-ordination relies on the synaptic vesicle priming factor UNC-13 and requires functional IRE-1 in the distal tissue.

**Figure 1:**
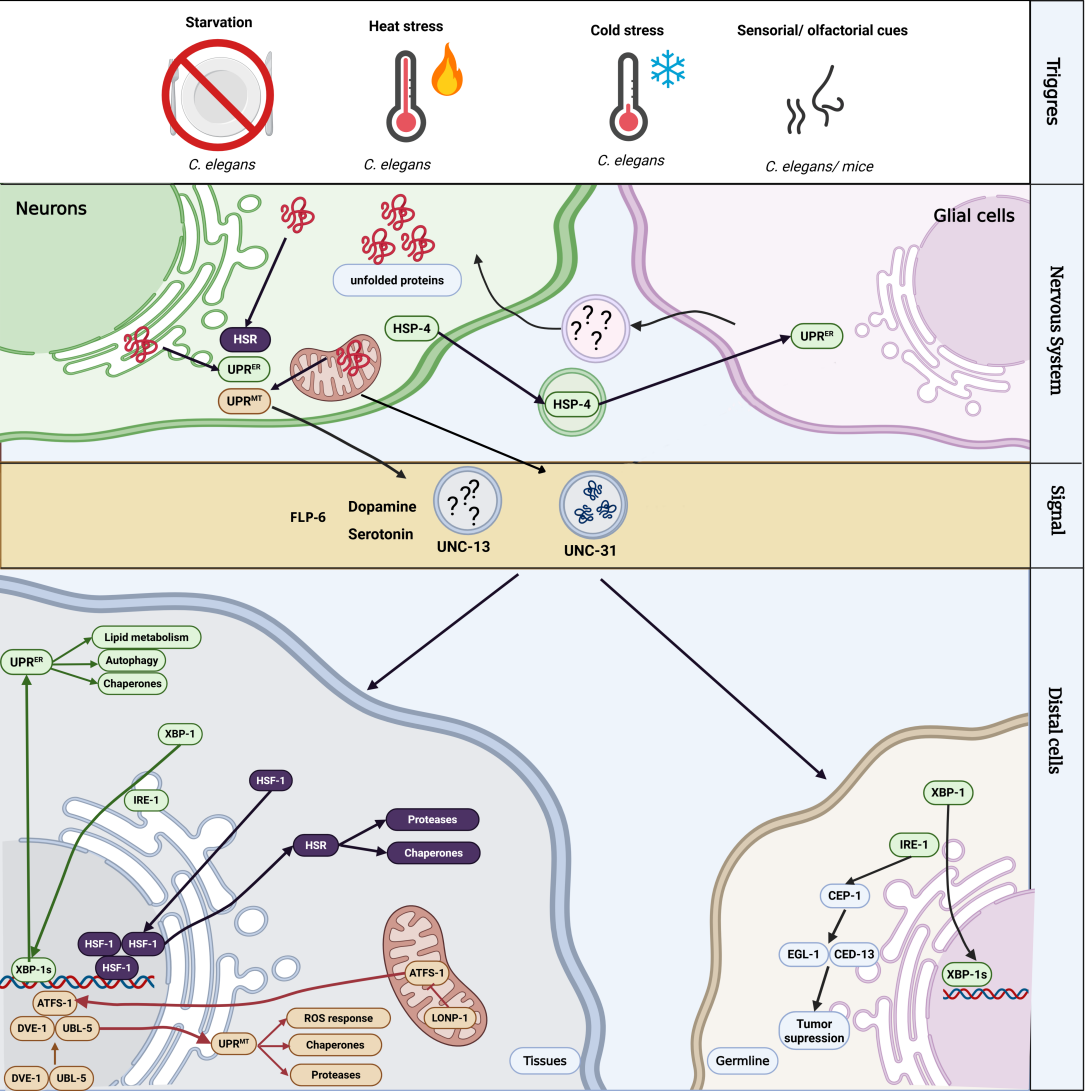
Cell-non-autonomous activation of the unfolded protein response of the endoplasmic reticulum (UPR^ER^), mitochondrial UPR (UPR^mt^), and heat shock response (HSR) pathways. Various stimuli such as thermal stress (heat or cold), odor perception, and starvation can trigger neuronal activation of stress responses in the nervous system of *C. elegans*. Similarly, in mammals, the sensory perception of food can also trigger cell-non-autonomous UPR^ER^ activation in the liver, although it remains unclear whether this signaling is mediated by visual cues, olfactory cues, or both [45]. Upon stimulus detection, neurons and glial cells activate stress-responsive transcriptional programs (UPR^ER^, UPR^mt^, and HSR) and secrete neuropeptides and neurotransmitters that mediate systemic signaling. These secreted factors – released through vesicular machinery involving UNC-31, UNC-13, and specific neuropeptides such as FLP-6 – co-ordinate stress responses in distal tissues. FLP-6-mediated germline apoptosis arises from neuronal IRE-1 activation. Target tissues respond by activating protective programs, including up-regulation of chaperones, proteases, autophagy, and lipid metabolism genes, thereby promoting organismal proteostasis and stress resilience. Question marks indicate mediators whose identities are still unknown. Created with BioRender.com.

Thus, cell-non-autonomous stress signaling refers to the capacity of cells – most often within the nervous system – to co-ordinate adaptive responses in distant tissues (Figure 1). This signaling plays important roles in modulating key physiological processes such as thermal adaptation, metabolism, development, reproduction, and immunity [46]. For example, through anticipatory mechanisms, neurons can fine-tune proteostasis networks systemically, preparing the organism for environmental or internal challenges [47–49]. Below, we will discuss how the HSR and the UPRs are regulated in distal tissues by neural cells across different physiological contexts.

### Thermal adaptation

Adapting to extreme temperatures is essential for surviving challenging environments and requires co-ordination between neuronal sensing and systemic stress responses. In *C. elegans*, heat stress is initially detected by AFD thermosensory neurons, which transduce thermal cues into organism-wide activation of the HSR through the transcription factor HSF-1 [43]. Importantly, neural activity is sufficient to trigger HSF-1-dependent transcription in distal tissues, inducing chaperone expression and enhancing thermotolerance even in the absence of direct heat exposure [50,51].

Optogenetic activation of AFD neurons is enough to induce HSF-1 nuclear localization in germline nuclei and drive *hsp-70* transcription [51]. This neuroendocrine response required serotonin as the inhibition of serotonin biosynthesis abolishes distal HSF-1 activation. The serotonin receptor SER-1 (a G-protein-coupled receptor [GPCR] homologous to mammalian 5-HT₂) is required for this response, since *ser-1* mutants fail to activate HSR in a cell-non-autonomous way [51]. These findings establish a circuit where AFD neurons, via serotonin/SER-1 signaling, propagate stress cues to peripheral tissues, coupling sensory detection with proteostasis programs.

Beyond chaperone induction, neuronal HSF-1 co-ordinates metabolic adaptation. In *C. elegans*, activation of neuronal HSF-1 promotes systemic lipid desaturation, adjusting membrane composition to sustain fluidity and maintain organismal thermotolerance [52]. A parallel mechanism has been described for cold adaptation. Neuronal activation of the ER stress sensor IRE-1 in sensory neurons induces expression of desaturase enzymes, increasing unsaturated fatty acid synthesis across tissues and thereby preserving membrane dynamics at low temperatures [53]. Interestingly, mild hypothermia in cortical neurons derived from human pluripotent stem cells activates the UPR^ER^, protecting cells against tunicamycin-induced ER stress [54]. Thus, it is tempting to think that neuroendocrine regulation may represent an evolutionarily conserved strategy to couple environmental sensing with systemic stress adaptation.

### Metabolism regulation

Mounting evidence supports the idea that neuronal stress responses and lipid metabolism regulation are intertwined. Constitutive neuronal expression of XBP-1s induces metabolic remodeling, including reduced triglycerides and elevated oleic acid [55] *–* a monounsaturated fatty acid linked to lifespan extension in *C. elegans* [56]*.* This remodeling depends on lysosomal lipases and up-regulation of the Δ9 desaturase FAT-6, an enzyme essential for OA biosynthesis [55]. Neuronal XBP-1s-induced lipid depletion also involves lipophagy mediated by the RAB-10/EHBP-1/RME-1 complex [57]. Interestingly, glial expression of XBP-1s can elicit similar lipid mobilization in peripheral tissues [58], indicating that non-neuronal cells in the nervous system can also exert systemic control over metabolism via proteostasis-related pathways activation*.*

Studies in *C. elegans* identified two distinct neuronal circuits that independently mediate cell-non-autonomous UPR^ER^ outputs: serotonergic neurons driving proteostasis-related genes and dopaminergic neurons regulating lipid metabolism [59]. Overexpression of *xbp-1s* in serotonergic neurons was sufficient to activate the intestinal cells, leading to the up-regulation of the ER chaperone *hsp-4* and calreticulin *crt-1*. This serotonergic UPR^ER^ axis promoted enhanced resistance to ER stress and modestly extended lifespan, both effects dependent on serotonin biosynthesis and *xbp-1* function. Crucially, increasing serotonin signaling alone, without XBP1s expression, did not activate UPR^ER^ [59], raising the possibility that transcriptional reprogramming within serotonergic neurons, rather than neurotransmitter release alone, is essential for initiating the cascade. In contrast, dopaminergic overexpression of *xbp-1s* did not activate the expression of chaperones but triggered peripheral lipophagy, driven by dopamine signaling and EH domain-binding protein 1 (EHBP-1) activation.

Some of these mechanisms seem to be conserved in mammals. In mice, the expression of Xbp1s in hypothalamic pro-opiomelanocortin (POMC) neurons improves systemic insulin sensitivity and reduces hepatic gluconeogenesis, even under conditions of diet-induced obesity [60]. Notably, constitutive Xbp1s expression in POMC neurons triggers transcriptional up-regulation of Xbp1s and its downstream target genes (e.g. Edem1, Erdj4, Chop) in the liver [60]. Furthermore, food-associated olfactory and visual stimuli can also activate hepatic XBP1s via hypothalamic circuits and through norepinephrine signaling [45], further supporting the idea that cell-non-autonomous regulation of the proteostasis network is a conserved feature of mammalian physiology. A recent work demonstrated that obesity impairs the IRE1-XBP1 branch of the UPR^ER^ in the anterior pituitary of mice, leading to defective hormone secretion, disrupting systemic thyroid hormone signaling [61]. This endocrine defect compromises hepatic XBP1 activity and the adaptive UPR^ER^ in the liver, promoting steatosis. Restoration of pituitary UPR^ER^ reactivates hepatic UPR function and alleviates metabolic dysfunction, establishing a pituitary–liver UPR^ER^ axis critical for metabolic regulation.

Cell-non-autonomous UPR^ER^ signaling has a relevant role in the regulation of systemic lipid metabolism. For instance, ceramides, bioactive lipids synthesized under ER stress, have been identified as paracrine signals that propagate UPR^ER^ activation to other cells. Palmitate-induced ER stress in skeletal muscle cells promotes the secretion of long-chain ceramides (C40:1 and C42:1), which, when internalized by naive myotubes, activate the UPR^ER^ via PERK signaling and dihydroceramide accumulation [62]. This raises the intriguing possibility that ceramides could act as lipid-based endocrine signals that extend the impact of localized ER stress to other tissues, potentially influencing whole-body energy homeostasis and contributing to metabolic disorders such as type 2 diabetes and obesity [63].

### Development and reproduction

Under stress, neurons can modulate development and reproduction to reallocate resources toward somatic survival. In *C. elegans*, ER stress in the ASI sensory neurons activates the ER stress sensor IRE-1, which cell-non-autonomously triggers germline apoptosis through the canonical apoptotic machinery, requiring CEP-1/p53 and CED-3/4 but independent of XBP-1 [64]. Beyond apoptosis, neuronal IRE-1 also modulates germline tumor fate. In the context of *gld-1*-deficient tumorous germlines, ER stress-induced IRE-1 activity promotes germ cell transdifferentiation into somatic-like cells, restoring their apoptotic competence and thereby suppressing tumor progression [65]. This effect relies on IRE-1-mediated degradation (RIDD) of *flp-6* mRNA in ASE neurons. *flp-6* encodes a neuropeptide that normally sustains a neuronal circuit preventing germline cells from differentiating. When FLP-6 is lost, this inhibitory circuit collapses, and germ cells inappropriately adopt somatic fates [66].

During periods of environmental stress, *C. elegans* also possesses the ability to modulate its overall reproductive status through the modulation of the UPR^ER^. Starved animals display more activation of nuclear XBP-1, and the neuronal overexpression of *xbp-1s* decreases brood size, potentially via a tyramine-mediated signaling pathway [67]. ER stress signaling in neurons also regulates the decision to enter the dauer stage, a quiescent developmental program that promotes survival under adverse conditions. In particular, PERK-mediated phosphorylation of eIF2α in ASI neurons triggers dauer by initiating downstream cell-non-autonomous signals [68].

### Immune response

Neurons also contribute to systemic immune priming through sensory perception of pathogen-associated cues. In *C. elegans*, chemosensory detection of volatile pathogen-associated molecules can initiate UPR^ER^ and UPR^mt^ signaling in peripheral tissues, along with innate immune activation [47,48,69]. Interestingly, *daf-7* (the *C. elegans* homolog of TGF-β) has been implicated in both UPR^ER^ and UPR^mt^ cell-non-autonomous signaling [48,70]. Notably, it was shown to be required for the former but suppresses the latter. Some of these responses can also be modulated by neuronal receptors and adaptors such as OCTR-1 and ARR-1. In ASH and ASI neurons, OCTR-1, an octopamine GPCR, suppresses IRE-1/XBP-1 branch of the UPR^ER^, enabling PMK-1/p38 MAPK activation [71,72], while ARR-1 modulates UPR^ER^ across a broader set of sensory neurons (e.g. ASH, ASI, AFD, and ADF) [73].

Cell-non-autonomous UPR^mt^ signaling is likewise critical for immune defense. Neuronal knockdown of *fzo-1*, a mitofusin, activates UPR^mt^ and induces mitochondrial fragmentation in peripheral tissues, which correlates with improved resistance to *Pseudomonas aeruginosa* [74]. Additionally, UPR^mt^ activation in the intestine, triggered by the GPCR SRZ-75 and Gαq signaling in ADL neurons, promotes immune gene expression, reduces mitochondrial respiration, and alters lipid accumulation [75].

Furthermore, exposure to certain bacterial metabolites, such as 2,3-pentanedione, can influence mitochondrial dynamics via olfactory circuits, illustrating how olfactory sensing of environmental cues prepares the organism for potential metabolic or pathogenic stress [76]. Intriguingly, ablation of AWC neurons, the primary olfactory sensory neurons in *C. elegans* that detect volatile odorants, leads to robust activation of the UPR_mt_ in peripheral tissues and increased resistance to *P. aeruginosa* [*76*]*.*

Altogether, these findings show that neuronal sensing of environmental cues can drive stress response pathways activation in distal tissues, linking sensory circuits to systemic stress resistance. In the future, it will be very interesting to investigate to what extent other components of the proteostasis network can initiate cell-non-autonomous responses. For example, overexpression of the proteasome β-subunit *pbs-5* was recently shown to boost proteasome activity in muscle in an *unc-13*-dependent manner [77]. These findings raise the possibility that distinct arms of the proteostasis machinery, including the proteasome, autophagy, and chaperone systems, could act as inter-tissue communication hubs. Exploring how these pathways are co-ordinated across organs and integrated with environmental and metabolic cues will be crucial to understand how organismal proteostasis is maintained and how it declines during aging.

Moreover, the findings from invertebrate models demonstrate that proteostasis is not limited to being a cell-intrinsic property, as it can be co-ordinated by systemic communication between neurons, glia, and peripheral tissues. Yet, the ultimate relevance of these mechanisms lies in their potential contribution to organismal decline with age and to the onset of chronic diseases. While the molecular circuitry of stress responses has been dissected in simple models, it is during aging and in neurodegenerative disease that the consequences of failing proteostasis become most apparent. For this reason, the following sections turn to how these pathways intersect with mammalian physiology, highlighting both their dysregulation in age-related decline and their potential as therapeutic targets.

## Dysregulation and therapeutic targeting of adaptive intertissue proteostasis networks in aging and neurodegeneration

### Aging

**Figure 2:**
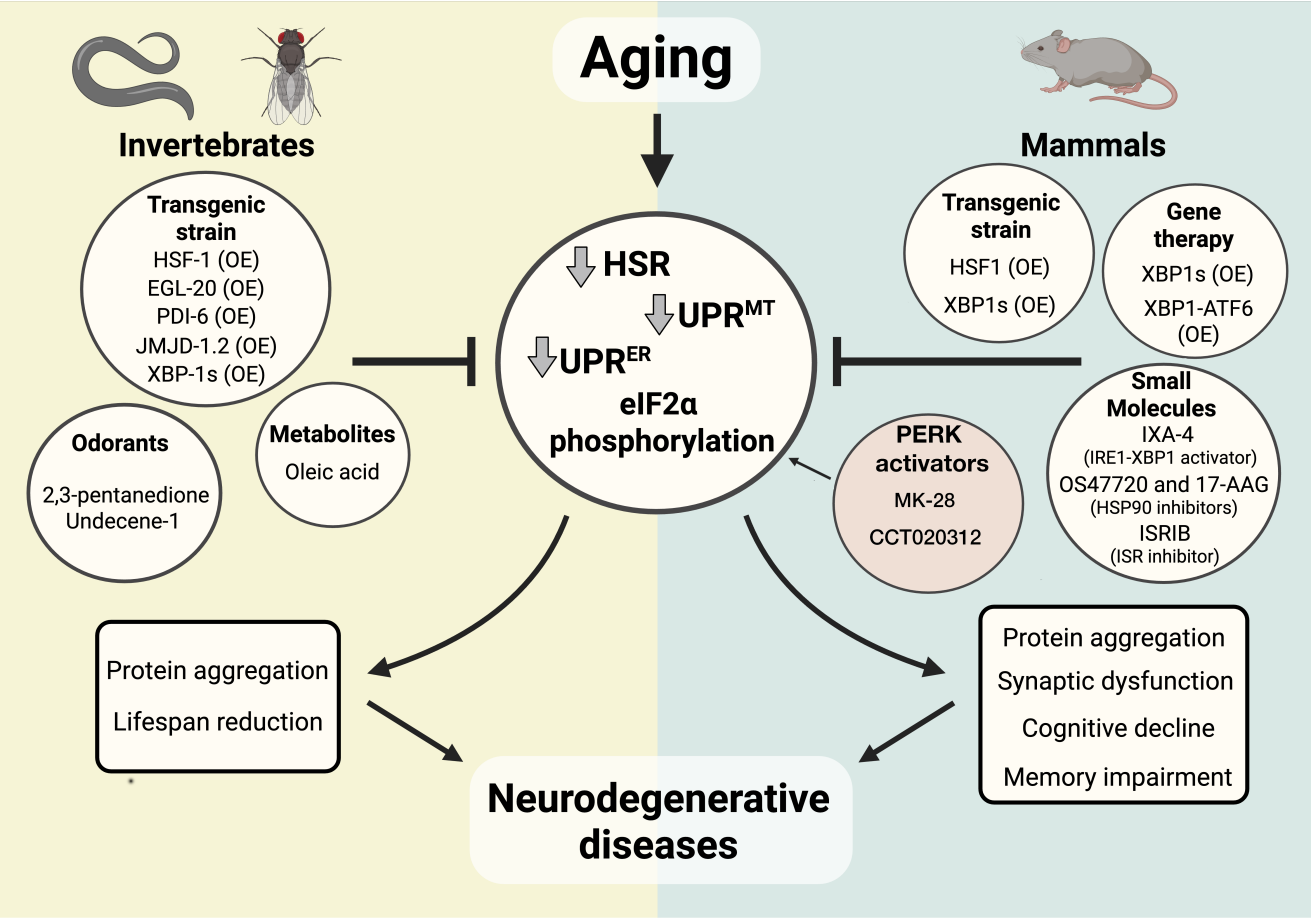
Proteostasis stress responses in aging and neurodegeneration. Aging alters the activity of key stress response pathways that maintain proteostasis, including the heat shock response (HSR), endoplasmic reticulum unfolded protein response (UPR^ER^), mitochondrial UPR (UPR^mt^), and eIF2α signaling [3,15,79]. These changes contribute to protein aggregation and increased vulnerability to neurodegenerative diseases. The decline of these pathways seems to involve chromatin and histone alterations that affect the activity of transcription factors such as HSF-1 in the HSR [15]. Specifically, the UPR^ER^ decline seems to be related to a decrease in the IRE1 RNAse activity as well, which disturbs the XBP1 splicing and regulated IRE1-dependent decay (RIDD) activities [80,81]. Invertebrate models have identified genetic and chemical modulators of these pathways, while studies in mammals highlight interventions through transgenic strains, small molecules, and gene therapy. Such strategies aim to restore proteostasis and mitigate cognitive decline, memory impairment, and synaptic dysfunction. Created with BioRender.com.

The capacity of neurons and glia to regulate proteostasis in distal tissues has implications for aging and disease. Building on this, recent studies have begun to explore how modulating these pathways can be harnessed for therapeutic benefit. A growing body of evidence demonstrates that manipulating the UPRs and the HSR, including through cell-non-autonomous mechanisms, enhances the ability to maintain protein homeostasis and directly influences aging rates and susceptibility to age-related diseases [78] (Figure 2). These stress pathways, including the UPR^mt^, the UPR^ER^, and the HSR, are not only essential for cellular function but also operate through systemic signaling, particularly in invertebrates, to co-ordinate organismal responses to proteotoxic stress.

In a seminal study, Durieux and colleagues demonstrated that knocking down mitochondrial, ETC. complex IV subunit *cco-1* in neurons activated the UPR^mt^ in distal intestinal cells and significantly extended *C. elegans* lifespan [44]. Follow-up studies revealed that this cell-non-autonomous UPR^mt^ activation requires neuropeptide signaling and Wnt pathway components, particularly signaling through the retromer complex [82,83]. Notably, neuronal overexpression of the Wnt ligand EGL-20 is sufficient to extend lifespan of *C. elegans* [83]. Further mechanistic insights identified the protein disulfide isomerase PDI-6 as a key regulator in this process. PDI-6, whose levels decline with age, modulates EGL-20 signaling in neurons and is essential for the cell-non-autonomous induction of UPR^mt^. Its overexpression is sufficient to extend lifespan in a UPR^mt^-dependent manner [84].

Emerging evidence highlights the active role of glial cells in UPR^mt^ signaling. A recent study revealed that inducing UPR^mt^ specifically in the four cephalic sheath (CEPsh) glia through the local overexpression of the histone demethylase JMJD-1.2 extends lifespan, enhances oxidative stress resistance, and reduces polyglutamine aggregation in *C. elegans* [85]. The proposed model implicates a glia-to-neuron signal, with neurons subsequently relaying the signal to peripheral tissues using dense-core vesicles and Wnt ligands. Together, these findings support a model in which both neurons and glia serve as upstream regulators of systemic proteostasis through cell-non-autonomous UPR^mt^ activation.

In general, across species ranging from *C. elegans* to mammals, the ability to activate stress response decays over time [15,80,86], and this age-associated decline is not restricted to mitochondrial responses. Taylor and colleagues reported that young *C. elegans* display robust transcriptional activation of UPR^ER^ genes in response to ER stress, whereas aged animals exhibit a significantly blunted response [79]. While the precise molecular underpinnings of this decline remain to be fully elucidated, emerging evidence supports that distinct mechanisms might be occurring simultaneously. For example, alterations in chromatin states (i.e. H3K27 trimethylation) appear to repress stress-responsive gene activation by restricting transcriptional initiation at promoters and enhancers [15]. In this scenario, stress-activated transcription factors may fail to effectively access chromatin, leading to reduced induction of downstream adaptive genes. Second, posttranslational modifications to the level of stress sensors, such as IRE1, may attenuate their responsiveness. Recent work has shown a correlation of aging to increased S-nitrosylation of IRE1 in the brains of aged mice, concomitant with a decline in the splicing of XBP1 [80]. Complementary evidence comes from *C. elegans*, where *xbp-1* mRNA splicing and RIDD activity both decline at older animals [81]. Because *ire-1* mRNA and protein levels do not significantly decrease, the loss of function appears to stem from an impairment in IRE-1’s activation rather than simple down-regulation of expression. Remarkably, constitutive expression of the active spliced form of the UPR^ER^ transcription factor *xbp-1s* in neurons not only restored ER stress responses in aged animals but also extended lifespan via a cell-non-autonomous mechanism [79].

Neuronal activation of the UPR^ER^ has been shown to trigger a subsequent activation of the pathway in the intestine, which plays a central role in mediating the observed longevity benefits [55,79,87]. The overexpression of UPR^ER^ components specifically in the intestine is sufficient to extend lifespan in worms, highlighting the intestine as a critical effector tissue in this process. Curiously, activation of the UPR^ER^ throughout the entire body did not result in lifespan extension, and in the case of muscle-specific activation, it actually shortens lifespan [79]. The reasons for these tissue-specific effects remain unclear and represent an important open question in the field. In mammals, the UPR^ER^ can act as an adaptive mechanism, enhancing protein folding and maintaining muscle homeostasis during exercise [88]. However, under chronic stress or in muscular dystrophies, UPR^ER^ triggers inflammation, mitochondrial dysfunction, and cell death, which could contribute to muscle fiber damage [89].

Activation of the IRE-1-XBP1 branch of the UPR^ER^ also extended the lifespan of flies [90], and neuronal XBP-1s activation in mice reduces hippocampal cellular senescence and prevents age-associated cognitive decline [80]. Although previous studies have shown that both XBP1 activation in POMC neurons and food-related sensory perception can induce the UPR^ER^ in the liver through cell-non-autonomous mechanisms [45,60], it remains unclear whether this peripheral activation produces similarly beneficial effects in mammals – such as increased stress resistance and lifespan – as extensively reported in *C. elegans*. In *C. elegans*, neuronal activation of the UPR^ER^ triggered IRE-1/XBP-1 activation in distal tissues, particularly the intestine. Interestingly, this mechanism is dependent on UNC-13, a protein involved in the formation of clear synaptic vesicles, further supporting the existence of a neuronal secretory pathway that can induce peripheral UPR^ER^. While the identity of the molecular messenger remains unclear, tyramine-mediated signaling could be implicated [67].

Recent evidence points to a central role of glia in the regulation of systemic proteostasis. In *C. elegans*, *xbp-1s* expression in CEPsh glia – astrocyte-like cells in the nematode – was shown to activate the UPR^ER^ in distal intestinal cells and extend lifespan [91]. Unlike other forms of neuronally induced UPR^ER^ signaling, this glia-to-intestine response required UNC-31, but not UNC-13. UNC-31 is important for the release of dense-core vesicles, which is enriched for neuropeptides [92], suggesting that different signaling molecules could mediate distal UPR^ER^ activation in *C. elegans*. Interestingly, a recent study demonstrated that in *C. elegans,* the amphid channel neurons ASH and ADL release extracellular vesicles containing the ER chaperone HSP-4 (BiP), which are taken up by amphid sheath (AMsh) glia [93]. Within glia, neuronal HSP-4 activates the IRE-1-XBP-1 branch of the UPR^ER^, leading to increased expression of chondroitin synthases. Notably, how neuronal HSP-4 activates IRE-1 in glia remains unclear. One intriguing possibility is that HSP-4 is transferred carrying misfolded proteins from ASH/ADL neurons into AMsh glia, thereby indirectly stimulating UPR^ER^ activation. More broadly, extracellular vesicles may represent a conserved mechanism for propagating proteostatic stress beyond neuron–glia interactions, potentially also contributing to neuron–intestine signaling described in other contexts. Collectively, these studies illustrate that glia engage in multiple modes of proteostatic communication with neurons and peripheral tissues.

Complementing these glial pathways, neuronal XBP-1s signaling drives its own distinct metabolic and longevity programs. Overexpression of the lipophagy component EHBP-1, a key player in the neuronal XBP-1s downstream pathway, is sufficient to mimic the effects of neuronal XBP-1s activation, leading to lipid depletion, ER remodeling, and extended lifespan [57]. Furthermore, XBP-1s expression in neurons increases the levels of intestinal oleic acid. Dietary supplementation with oleic acid extends the lifespan of wildtype worms and enhances proteostasis but does not further extend the lifespan of worms overexpressing XBP-1s, suggesting that oleic acid acts downstream or in parallel with this pathway [55]. Imanikia and colleagues further demonstrated that neuronal *xbp-1s* promotes broad transcriptional changes in the intestine, notably up-regulating lysosomal genes such as *lmp-1*, *asp-3*, and *vha-18* [87]. This response enhances autolysosomal activity, promoting autophagosome maturation. The lysosomal biogenesis factor *hlh-30* (the worm ortholog of TFEB) is required for these effects and mediates both lysosomal gene induction and the longevity benefits of *xbp-1s* activation.

The HSR also plays a critical cell-non-autonomous role in aging. Overexpression of HSF-1, the master regulator of the HSR, in neurons leads to activation of the FOXO transcription factor DAF-16 in the intestine, promoting longevity independently of canonical thermosensory circuits [50]. Similarly, overexpression of HSF-1 in CEPsh glia cells induces the HSR in peripheral tissues and increases lifespan [94], reinforcing the emerging concept that glia, like neurons, can co-ordinate systemic stress responses.

### Neurodegenerative diseases

Because of their ability to influence organismal proteostasis, these stress response pathways, namely UPR^mt^, UPR^ER^, and HSR, have been investigated as potential therapeutic targets for neurodegenerative diseases characterized by proteotoxic stress, such as AD, PD, and HD [95]. Increasing HSF-1 expression by genetic manipulation ameliorates the burden caused by toxic protein aggregates. Overexpressing HSF-1 reduced polyglutamine (polyQ) aggregation in both worm, cell, and mouse models [96,97]. Moreover, transgenic overexpression of HSF-1 reduced the load of amyloid-beta (Aβ) plaques and ameliorated cognitive defects in a human amyloid precursor protein (PDAPP) mouse model of AD [98]. In agreement, the activation of HSF-1 by pharmacological inhibition of HSP90 by small molecules, such as OS47720 and 17-AAG/Tanespimycin, improved synaptic function and memory in AD mice model [99,100]. Notably, brain samples from AD and HD patients, as well as mouse models, show abnormal degradation of HSF-1, which may further compromise stress resilience in these conditions [101,102].

PolyQ-induced neuronal proteotoxic stress activates the UPR^mt^, which in turn disrupts mitochondrial function in peripheral tissues in *C. elegans* [103]. This response is conserved in human primary fibroblasts [103]. ATF5, a key regulator of the UPR^mt^, accumulates in polyQ nuclear inclusions and is depleted in HD mouse and human brains. While loss of ATF5 exacerbates polyQ toxicity in *C. elegans*, its overexpression reduces apoptosis in mammalian HD cell models, highlighting its neuroprotective potential [104].

Aβ itself can also activate the UPR^mt^ in mice and human cells [105], and inhibition of the UPR^mt^ aggravates Aβ-induced toxicity, while enhancement of mitochondrial proteostasis mitigates disease phenotypes [105,106]. The role of UPR^mt^ in PD progression, however, appears to be context-dependent. While moderate activation may be neuroprotective and promote dopaminergic neuron survival [107], excessive or chronic activation, especially via the transcription factor ATFS-1, can exacerbate pathology [108]. These findings emphasize that the magnitude and context of stress pathway activation are critical determinants of their therapeutic efficacy.

As mentioned, neuronal expression of *xbp-1s* enhances systemic proteostasis and facilitates the clearance of toxic aggregates such as Aβ and polyQ in *C. elegans*, hallmarks of AD and HD, respectively [87]. Interestingly, exposure to specific odorants, particularly 1-undecene, is sufficient to activate UPR^ER^ in peripheral tissues, resulting in less accumulation of polyQ aggregates in the muscle and increased lifespan [48]. The neuroprotective role of the UPR^ER^ has also been demonstrated in various models of Aβ toxicity. XBP-1s expression confers protection in *Drosophila* models of AD and in mammalian neurons exposed to Aβ oligomers [109]. Transgenic mice overexpressing XBP1s show reduced Aβ load and preserved cognitive function [110]. Furthermore, gene therapy delivering XBP1s to the hippocampus improves synaptic plasticity and mitigates cognitive decline in aged and AD mice [80,110]. Importantly, the protection promoted by XBP1s in a *Drosophila* model of AD was lost with aging, suggesting that these benefits may be restricted to an optimal time window [111].

Expression of XBP1s also demonstrates protective effects in PD models [112]. In human dopaminergic neuroblastoma cells, XBP1s overexpression reduces toxicity from PD-related insults, and adenoviral-mediated delivery of XBP1s in a mouse PD model significantly protects dopaminergic neurons from degeneration. Interestingly, enforced expression of an ATF6/XBP1s fusion protein via gene therapy mitigated neurodegeneration in HD and PD models more effectively than either transcription factor alone, highlighting the therapeutic potential of combining two major UPR^ER^ signaling branches [113]. Finally, novel molecules, such as IXA-4, that can pharmacologically activate the IRE1-XBP1 axis have been tested and been shown to improve phenotypes in the context of metabolic diseases and in a Charcot–Marie–Tooth type 1B neuropathy disease model in mice [114,115].

On the other hand, chronic activation of the ISR via PERK is implicated in age-related cognitive decline and neurodegeneration. Sustained eIF2α phosphorylation suppresses global translation, including essential synaptic proteins, thereby impairing memory consolidation and synaptic plasticity [116]. Notably, small molecules targeting the ISR have shown promise in reversing these effects. One such compound, integrated stress response inhibitor (ISRIB), acts downstream of eIF2α phosphorylation to restore translation without affecting upstream stress-sensing kinases [117,118]. In mice, ISRIB treatment counteracts the effects of eIF2α phosphorylation, improves memory and synaptic plasticity in AD models [119], and alleviates the age-related memory impairment in old animals [120]. Additionally, other compounds with ISRIB-like activity, such as trazodone hydrochloride and dibenzoylmethane, also promoted neuroprotection against prion disease and frontotemporal dementia in mice [121].

Importantly, however, recent evidence demonstrates that controlled or transient PERK activation may bolster cellular defenses and confer neuroprotection. PERK activators – including MK-28 and CCT020312 – reduced neuronal apoptosis, decreased pathological protein species, and mitigated neurodegeneration in Huntington’s and tauopathy models. In tauopathy models, the PERK activator CCT020312 decreased pathological tau phosphorylation and conformational changes, induced NRF2-dependent cytoprotective programs, rescued dendritic spines and motoneurons, and improved memory and motor performance [122]. Likewise, in HD models, MK-28 selectively activated PERK, increased eIF2α-P in brain, and improved motor and metabolic phenotypes, substantially extending lifespan in R6/2 mice [123,124]. These findings indicate that the therapeutic modulation of PERK is probably context-dependent, where activation may be beneficial in diseases characterized by insufficient stress signaling or specific aggregate loads.

## Conclusion and future perspectives

Proteostasis is maintained by a multifaceted and integrated cellular network encompassing HSR, UPR^ER^, UPR^mt^, among other pathways. Canonical mechanisms rely on chaperone-mediated stress sensing and transcriptional regulation to restore homeostasis. However, recent discoveries have uncovered a range of non-canonical regulatory layers, including lipid bilayer stress sensing, neuroendocrine communication, and inter-organelle signaling. These alternative modes of regulation not only enhance the adaptability of stress responses but also extend their influence across tissues through cell-non-autonomous mechanisms. As tools such as single-cell and spatial transcriptomics become more accessible, it will be possible to dissect how stress responses are orchestrated across tissues in real time.

Unraveling how neurons and glia communicate stress signals to peripheral tissues in higher organisms will be essential to understand the systemic nature of proteostasis collapse during aging. This is particularly urgent in the context of neurodegeneration, where disease progression may reflect not only cell-autonomous failure but also a breakdown of organism-wide co-ordination. Bridging mechanistic work in model organisms with mammalian disease contexts will therefore open new opportunities for interventions that strengthen proteostasis at the whole-organism level. Thus, a deeper understanding of how these canonical and non-canonical pathways intersect, both within individual cells and at the organismal level, holds promise for the development of novel interventions targeting age-related diseases, neurodegeneration, and metabolic disorders.

**Key directions in intercellular proteostasis control**

- **To what extent do cell-non-autonomous stress responses that regulate proteostasis occur in mammals?** While compelling evidence exists in invertebrate models, mammalian studies have largely emphasized cell-autonomous mechanisms related to proteostasis regulation. It remains unclear whether neurons and glia can engage comparable systemic circuits in mammals, and whether such responses influence organismal aging and disease progression.
- **What are the molecular mediators through which neurons regulate proteostasis in distal tissues?** While signaling molecules such as serotonin, tyramine, and specific neuropeptides have been implicated, a comprehensive understanding of the secreted factors and mechanisms involved remains lacking.
- **To what extent do glial cells contribute to systemic proteostasis regulation?** While most current research has focused on neurons, particularly hypothalamic populations such as POMC neurons, where expression of XBP1s has been shown to activate the UPR^ER^ in peripheral tissues like the liver, comparable studies in glial cells are lacking. Whether glial expression of XBP1s, for instance in astrocytes or microglia, can similarly elicit cell-non-autonomous UPR activation remains unknown.
- **What is the functional relevance of sensory-driven proteostasis regulation in mammals?** Studies in invertebrate models have demonstrated a strong link between environmental perception and systemic proteostasis. However, whether similar sensory circuits exist and can be harnessed in mammals for therapeutic purposes, particularly in the context of metabolic or neurodegenerative diseases, remains an open question.
- **How is the ER stress sensor IRE1 activated in response to distal or systemic signals?** Is its activation mediated solely by canonical mechanisms such as unfolded proteins in the ER lumen or lipid bilayer stress, or are there novel, cell-non-autonomous triggers yet to be identified?
- **Is cell-non-autonomous proteostasis regulation unidirectional, or does peripheral feedback modulate neuronal control?** The possibility that peripheral tissues send feedback signals back to the nervous system to fine-tune stress responses remains unexplored.
- **Are additional proteostasis pathways, such as the ribosome-associated quality control (RQC) system, subject to cell-non-autonomous regulation?** If so, how are these pathways co-ordinated across tissues, and what are the upstream signals involved?
- **What underlies the age-associated decline in stress-response activity?** Although upstream signals may still be present in aged organisms, the downstream transcriptional or posttranslational responsiveness appears blunted. The molecular basis for this diminished capacity is still poorly understood.

## Abbreviations

AD: Alzheimer’s disease
ATF6: activating transcription factor 6
ATFS-1: activating transcription factor associated with stress-1
Aβ: amyloid-beta
CEPsh glia: cephalic sheath glia
DCVs: dense-core vesicles
ER: endoplasmic reticulum
ETC: electron transport chain
GCN2: general control nonderepressible 2
HD: Huntington’s disease
HRI: heme-regulated inhibitor
HSF1: heat shock factor 1
HSP: heat shock protein
HSR: heat shock response
IRE1: inositol-requiring enzyme 1
ISR: integrated stress response
ISRIB: integrated stress response inhibitor
JMJD-1.2: Jumonji domain-containing 1.2
LBS: lipid bilayer stress
OA: Oleic Acid
PD: Parkinson’s disease
PDI: protein disulfide isomerase
PERK: PKR-like ER kinase
PKR: protein kinase R
POMC: pro-opiomelanocortin
PP2A: protein phosphatase 2A
RIDD: regulated IRE1-dependent decay
RQC: ribosome-associated quality control
TGF-β: transforming growth factor beta
UPR: unfolded protein response
XBP1: X-box binding protein 1
eIF2α: eukaryotic initiation factor 2 alpha
polyQ: polyglutamine
